# Suicidal ideation during treatment of depression with escitalopram and nortriptyline in Genome-Based Therapeutic Drugs for Depression (GENDEP): a clinical trial

**DOI:** 10.1186/1741-7015-7-60

**Published:** 2009-10-15

**Authors:** Nader Perroud, Rudolf Uher, Andrej Marusic, Marcella Rietschel, Ole Mors, Neven Henigsberg, Joanna Hauser, Wolfgang Maier, Daniel Souery, Anna Placentino, Aleksandra Szczepankiewicz, Lisbeth Jorgensen, Jana Strohmaier, Astrid Zobel, Caterina Giovannini, Amanda Elkin, Cerisse Gunasinghe, Joanna Gray, Desmond Campbell, Bhanu Gupta, Anne E Farmer, Peter McGuffin, Katherine J Aitchison

**Affiliations:** 1 MRC SGDP Centre, Institute of Psychiatry at King's College London, UK; 2 Institute of Public Health, Ljubljana, Slovenia; 3 Central Institute of Mental Health, Division of Genetic Epidemiology in Psychiatry, Mannheim, Germany; 4 Centre for Psychiatric Research, Aarhus University Hospital, Risskov, Denmark; 5 Croatian Institute for Brain Research, Medical School, University of Zagreb, Zagreb, Croatia; 6 Laboratory of Psychiatric Genetics, Department of Psychiatry, Poznan University of Medical Sciences, Poznan, Poland; 7 Department of Psychiatry, University of Bonn, Bonn, Germany; 8 Université Libre de Bruxelles, Erasme Academic Hospital, Department of Psychiatry, Brussels, Belgium; 9 Biological Psychiatry Unit and Dual Diagnosis Ward IRCCS, Centro San Giovanni di Dio, Brescia, Italy; 10 Mood Disorders Research Unit, Aarhus University Hospital, Risskov, Denmark

## Abstract

**Background:**

Suicidal thoughts and behaviours during antidepressant treatment, especially during the first weeks of treatment, have prompted warnings by regulatory bodies. The aim of the present study is to investigate the course and predictors of emergence and worsening of suicidal ideation during tricyclic antidepressant and serotonin reuptake inhibitor treatment.

**Methods:**

In a multicentre part-randomised open-label study, 811 adult patients with moderate to severe unipolar depression were allocated to flexible dosage of escitalopram or nortriptyline for 12 weeks. The suicidality items of three standard measures were integrated in a suicidal ideation score. Increases in this score were classified as treatment emergent suicidal ideation (TESI) or treatment worsening suicidal ideation (TWOSI) according to the absence or presence of suicidal ideation at baseline.

**Results:**

Suicidal ideation decreased during antidepressant treatment. Rates of TESI and TWOSI peaked in the fifth week. Severity of depression predicted TESI and TWOSI. In men, nortriptyline was associated with a 9.8-fold and 2.4-fold increase in TESI and TWOSI compared to escitalopram, respectively. Retirement and history of suicide attempts predicted TWOSI.

**Conclusion:**

Increases in suicidal ideation were associated with depression severity and decreased during antidepressant treatment. In men, treatment with escitalopram is associated with lower risk of suicidal ideation compared to nortriptyline. Clinicians should remain alert to suicidal ideation beyond the initial weeks of antidepressant treatment.

**Trial registration:**

EudraCT (No.2004-001723-38) and ISRCTN (No. 03693000).

## Background

Every year more than 100,000 men and women commit suicide in Europe [1]. Mental disorders, especially depression, are present in more than 90% of suicides, and over 80% are untreated at the time of death [2,3]. While it is known that antidepressant medication alleviates depression, recent randomised controlled trials (RCTs) in adults have left the verdict open as to whether antidepressants reduce suicidal thoughts and behaviours [4,5]. Reports of higher rates of suicide-related adverse events during treatment with selective serotonin reuptake inhibitors (SSRIs) and other antidepressants compared to placebo have prompted regulatory bodies in the US and Europe to issue warnings alerting clinicians to the risk of suicide during the first weeks of antidepressant treatment [6,7]. These warnings have had an impact on prescribing and may have paradoxically increased the risk of suicide in the population [8].

The incidence of treatment emergent suicidal ideation (TESI) varies from 4% to 20%, depending on the definition of suicidal ideation and sample characteristics [9-13]. The risk has been reported to be highest within the first week of treatment or after an alteration of dose [14]. Several risk factors have been proposed, including severity of depression, younger age at onset, younger adults [11,13] and genetic factors [9,12]. Male gender has also recently been associated with worsening of suicidal ideation during citalopram treatment [15]. As there are controversies about gender differences in antidepressant treatment response, it is warranted to investigate moderation of suicidal ideation during such treatment by gender [16]. Perhaps the most clinically relevant, but as yet unanswered, question is whether a specific type of antidepressant (for example, SSRIs or tricyclic antidepressants (TCAs)) is associated with higher risk of TESI [4,13,14,17,18]. The Genome-Based Therapeutic Drugs for Depression (GENDEP) trial, the largest comparative study of an SSRI and TCA, is well suited to address this question.

Several previous investigations have been restricted to the emergence of suicidal ideation in individuals who reported no suicidal ideation at baseline. Whilst this approach highlights the relatively rare cases with *de novo *treatment emergent suicidal ideation, the potential for worsening of existing suicidal ideation during treatment with antidepressants remains relatively unexplored, thus risking 'throwing the baby out with the bath water' through removing a large proportion of patients at risk. Several other limitations could be raised concerning the measure of emergence of suicidal ideation in previous investigations. Firstly, most of these studies used only one scale to measure suicidal ideation: a more sensitive self-report questionnaire, risking false positives [9,12] or a more specific clinician-rated scale [10,11,13] risking false negatives. Secondarily, by using only one scale, a lot of missing values are encountered during the follow-up leading to loss of data. Thirdly, some of the investigations did not have repeated measures of suicidal ideation across the treatment period and only focused on the first weeks of treatment, potentially missing important information on the course of suicidal ideation [9,12]. Therefore, we here report, in a secondary analysis of GENDEP, the course and predictors of both emergence and worsening of suicidal ideation over 12 weeks of antidepressant treatment. Moreover, in order to use all available data and provide unbiased estimates in the presence of missing values, we apply the item response theory (IRT) [19,20] to derive the best estimate of suicidal ideation from the suicidality items of three rating scales: one self-report questionnaire and two clinician-rated scales.

## Methods

### Sample and study design

The sample comprised 811 subjects with major depressive disorder taking part in GENDEP, a partially randomised multicentre clinical and pharmacogenomic study [21]. Participants were included if they met criteria for a major depressive episode of at least moderate severity, as defined by the Diagnostic and Statistical Manual, fourth edition (DSM-IV) and International Classification of Diseases, 10th revision (ICD-10) criteria, established using the Schedules for Clinical Assessment in Neuropsychiatry (SCAN, version 2.1) [22]. Patients with no contraindications were randomly allocated to receive flexible dosage nortriptyline (50 to 150 mg daily) or escitalopram (10 to 30 mg daily) for 12 weeks. Patients with contraindications for one of the drugs were allocated non-randomly to the other antidepressant. Participants who could not tolerate the initially allocated medication or who did not experience sufficient improvement despite adequate dosage for 8 weeks were offered a change to the other medication. Participants who changed medication were then followed-up using the same protocol as for the first antidepressant. All subjects were of European ethnicity and between 18 and 72 years of age. The exclusion criteria were: a first-degree relative with bipolar affective disorder or schizophrenia, a history of hypomanic or manic episode, mood incongruent psychotic symptoms, primary substance misuse or primary organic disease, current treatment with an antipsychotic or a mood stabilizer, and pregnancy or lactation. A total of 15 subjects who had missing data on all three suicidality items at baseline were excluded.

The study protocol was approved by research ethics committees in each centre. After describing the study to the subjects, written informed consent was obtained. The GENDEP trial is registered at EudraCT (no. 2004-001723-38) and ISRCTN (no. 03693000).

### Severity of depressive disorder

Severity of depressive disorder was based on the 'observed mood' dimension derived by factor analysis of categorical items form three rating scales and scored through an item response theory procedure [20]. The 'observed mood' dimension is a highly internally consistent measure, comprising symptoms of depressed mood, activity, anxiety and psychomotor disturbance rated by the clinician. It contains information from items that constitute the previously suggested 'core' subscales of the Hamilton Rating Scale for Depression (HRSD) [23], and is therefore suitable for testing hypotheses related to pharmacological modulation of mood. Most importantly, this dimension does not contain any information from the suicidality items and thus there is no overlap of content with the suicidal ideation measures. For this reason, observed mood is suitable as a covariate in models that test suicidal ideation as the outcome of interest.

### Suicidal ideation

Suicidal ideation was assessed using a composite score based on the 3rd item of the 17-item HRSD (HRSD-17), the 9th item of the self-report 21-item Beck Depression Inventory (BDI) and the 10th item of the clinician-rated 10-item Montgomery-Asberg Depression Rating Scale (MADRS). The response options for these items are shown in Table 1.

**Table 1:** Range of response options for HDSR-17, MADRS and BDI suicide items

**Scale**	**Score**	**Meaning**
HRSD-17	0	Absent
	1	Feels life is not worth living
	2	Wishes he/she were dead, or any thought of possible death to self
	3	Suicide ideas or half-hearted attempt
	4	Attempts suicide
MADRS	0 to 1	Enjoys life or take it as it comes
	2 to 3	Weary of life. Only fleeting suicidal thoughts.
	4 to 5	Probably better off dead. Suicidal thoughts are common, and suicide is considered as a possible solution, but without specific plans or intentions.
	6	Explicit plans for suicide when there is an opportunity. Active preparation of suicide
BDI	0	Absent
	1	Thought of killing myself
	2	I would like to kill myself
	3	I would like to kill myself if I had a chance

BDI = Beck Depression Inventory; HRSD = Hamilton Rating Scale for Depression; MADRS = Montgomery-Asberg Depression Rating Scale.

As there is imperfect correspondence in the content and number of response options between these three scales, and as no scale had been proven to be optimal by itself to measure depression severity or suicidal ideation [20,24], we used the item response theory (IRT) graded response model to derive the best estimate of suicidal ideation from the suicidal items of the three rating scales. The IRT scoring allows the use of all available data and provides unbiased estimates in the presence of missing values [19,20].

The IRT-derived standardised composite score (θ) of suicidal ideation (ranging from -0.425 to 3.241) correlated 0.97, 0.92 and 0.77 with MADRS, HRSD-17, and BDI suicidal items, respectively. The discrimination (α) and threshold (β) parameters for the suicidal items are given in Table 2. The discrimination parameter α indexes an item's ability to differentiate between levels of suicidal ideation and the threshold parameters (β) are estimated points of transitions between subsequent response options on a latent continuous dimension of suicidal ideation, in units of 1 standard deviation (SD). Of note, the threshold of the first non-zero response option was 0.64 on BDI, 0.03 on MADRS, and 0.4 on HRSD-17, indicating that BDI was not only less discriminative but also less sensitive than the clinician-rated measures (Table 2).

**Table 2:** Item response characteristics for the three measures of suicidality

	**Discrimination parameter (α)**	**Response option thresholds**					
		
		**β1**	**β2**	**β3**	**β4**	**β5**	**β6**
MADRS suicidality	7.1	0.03	0.8	1.53	1.93	2.57	2.97
HRSD-17 suicidality	6.1	0.4	1.26	1.99	3.11		
BDI suicidality	3.1	0.64	1.98	2.66			

The threshold parameter β shows the standardised level of severity at which subsequent response options become more likely than the previous response option.

BDI = Beck Depression Inventory; HRSD = Hamilton Rating Scale for Depression; MADRS = Montgomery-Asberg Depression Rating Scale.

We defined significant suicidal ideation as an IRT score of at least 1 SD above 0 (0 scores on all three scales corresponded to -0.425 on the standardised IRT θ scale; thus 0.575 or more is considered as significant suicidal ideation). This threshold corresponds to the 75th percentile of baseline scores and captures individuals reporting significant suicidal ideation (score of 1 or more on BDI and HRSD-17; 2 or more on MADRS) on at least two of the three scales. This is consistent with previous definitions of treatment-emergent suicidal ideation using the 'thoughts of death or suicide' as a threshold (Table 3). Based on this definition, 473 participants reported significant suicidal ideation at baseline. Most of them (67%) reported suicidal ideation on all three measures.

**Table 3:** Equivalent summed item response theory score estimates for subjects without suicidal ideation at the baseline (NSI) and subjects with suicidal ideation at the baseline (SI)

**NSI**						**SI**					
**N**	**%**	**θ**	**MADRS**	**HRSD-17**	**BDI**	**n**	**%**	**θ**	**MADRS**	**HRSD-17**	**BDI**

108	33.4	-0.425	0	0	0	3	0.6	0.613	0	2	1
7	2.2	-0.392	0	0	-	1	0.2	0.626	2	0	-
1	0.3	-0.27	-	0	0	44	9.3	0.655	1	1	1
2	0.6	-0.173	1	0	1	8	1.7	0.707	2	0	1
15	4.6	-0.058	0	0	1	2	0.4	0.742	1	1	2
3	0.9	-0.036	0	0	2	1	0.2	0.746	1	1	3
6	1.9	0.114	0	1	0	2	0.4	0.775	1	2	0
55	17.0	0.163	1	0	0	1	0.2	0.809	2	0	2
6	1.9	0.196	1	0	-	46	9.7	0.845	2	1	0
2	0.6	0.259	1	-	0	2	0.4	0.849	2	-	0
7	2.2	0.288	0	1	1	2	0.4	0.885	-	-	1
18	5.6	0.331	1	0	1	7	1.5	0.943	1	2	1
1	0.3	0.353	1	-	-	5	1.1	0.962	2	1	-
84	26.0	0.494	1	1	0	79	16.7	0.999	2	1	1
2	0.6	0.539	2	0	0	1	0.2	1.076	2	-	1
6	1.9	0.569	1	1	-	3	0.6	1.14	2	1	2
						
						2	0.4	1.153	2	1	3
						9	1.9	1.172	2	2	0
						3	0.6	1.206	3	1	0
						63	13.3	1.317	2	2	1
						5	1.1	1.318	2	2	-
						7	1.5	1.356	3	1	1
						7	1.5	1.444	2	2	2
						4	0.9	1.467	2	2	3
						2	0.4	1.483	3	1	2
						1	0.2	1.492	4	1	1
						4	0.9	1.501	3	2	0
						5	1.1	1.548	2	3	1
						1	0.2	1.572	2	4	1
						50	10.6	1.602	3	2	1
						4	0.9	1.627	3	2	-
						12	2.5	1.713	3	2	2
						2	0.4	1.751	3	2	3
						1	0.2	1.774	2	3	3
						9	1.9	1.827	3	3	1
						17	3.6	1.841	4	2	1
						1	0.2	1.893	4	2	-
						2	0.4	1.928	3	3	2
						8	1.7	1.95	4	2	2
						2	0.4	1.983	3	3	3
						1	0.2	2.01	4	2	3
						11	2.3	2.079	4	3	1
						1	0.2	2.172	4	3	-
						13	2.8	2.199	4	3	2
						2	0.4	2.324	4	3	3
						1	0.2	2.325	4	4	0
						1	0.2	2.387	4	4	1
						2	0.4	2.453	5	3	1
						1	0.2	2.515	4	4	2
						1	0.2	2.553	5	3	2
						4	0.9	2.677	5	3	3
						1	0.2	2.911	6	3	-
						2	0.4	2.954	6	3	3
						1	0.2	3.086	6	4	2
						1	0.2	3.106	-	4	-
						2	0.4	3.241	6	4	3

BDI = Beck Depression Inventory; HRSD = Hamilton Rating Scale for Depression; MADRS = Montgomery-Asberg Depression Rating Scale.

TESI was defined as any increase above the threshold (θ = 0.575) at any point during treatment in individuals without suicidal ideation at baseline. To avoid classifying individuals as TESI when their IRT score increased minimally due to variation in the proportion of missing data, an additional criterion of an increase of at least 0.5 SD was employed.

Treatment worsening suicidal ideation (TWOSI) was defined as an increase of suicidal ideation of at least 0.5 SD above the individual's baseline score at any point during the study in individuals with significant suicidal ideation at baseline.

### Statistical analysis

We used a generalised linear latent and mixed model (GLLAMM) in Stata (Stata, College Station, TX, USA) with adaptive quadrature to obtain maximum likelihood estimates for the individual random intercept and slope model and to assess clinical and demographic predictors of TESI and TWOSI [25]. The strength of the maximum likelihood estimation is that no information is lost and it is robust to missing data. To relax the assumption of conditional independence in the responses of the same person and for the same centre of recruitment, we included a subject-specific random intercept nested in a centre-specific random intercept. This method provides more accurate measures of confidence intervals than traditional logistic models, which tend to underestimate uncertainty if data, as in this instance, have a hierarchical structure. Subjects who changed medication were included under both medications with individual-level clustering being controlled by the random effect of individual. The analyses assumed a binomial error distribution. Potential predictors were included as fixed factors, change in drug dosage and depression severity were included as time-varying predictors. Age was firstly analysed as a continuous variable and secondarily as a categorical variable according to US Federal Drug Administration (FDA) recommendations: lower than 25 years old versus 25 years or older [7]. Interactions between gender, age and drug were also tested.

Three comparisons were made: (i) any increase in suicidal ideation (TESI/TWOSI) versus no increase in the whole sample, (ii) TESI versus no TESI among subjects without suicidal ideation at baseline, (iii) TWOSI versus no TWOSI among subjects with suicidal ideation at baseline.

Two sensitivity analyses were conducted to test the robustness of results. First, to check whether results hold for larger increases in suicidal ideation, we repeated all analyses with a higher threshold of 1 SD increase in suicidal ideation. Second, to exclude the influence of selection bias inherent in non-random allocation to drug in a proportion of the sample, we repeated the analyses in a reduced sample of individuals who were randomly allocated. In the latter set of sensitivity analyses, individuals who switched from the randomly allocated medication were considered as missing after the switch.

Mixed linear models as described elsewhere [20] were used to measure the effect of treatment on the continuous suicidal score over time. A two-tailed *P *value < 0.05 was considered as statistically significant. All analyses were conducted in Stata, version 10.

### Power calculation

The overall sample had 97% power to detect a significant difference between the two drugs at an α level of 0.05 assuming a baseline rate of 10% and an increase of 10% for the drug associated with higher risk of increase in suicidal ideation. Power to detect interactions was calculated using Quanto software [26]. We calculated that we had 56% and 78% power to detect an interaction between drug and gender and between drug and age assuming an odds ratio (OR) of 2 or more.

## Results

### Sample baseline characteristics

**Figure 1:**
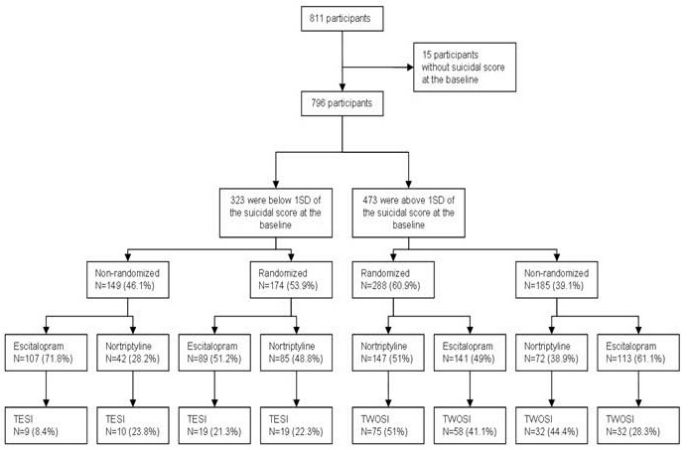
**Flow of participants throughout the study**.

Figure 1 displays the flow of participants throughout the study. The baseline demographic and clinical characteristics of the 323 non-suicidal subjects and the 473 suicidal subjects are shown in Table 4. Subjects who reported suicidal ideation at baseline had more severe depression (0.75 (0.54) versus 1.06 (0.59), *P *< 0.0001). More subjects with suicidal ideation at baseline reported a history of suicide attempts than individuals without suicidal ideation at baseline (19.3% versus 6.3%, *P *< 0.0001).

**Table 4:** Sociodemographic and clinical characteristics of participants without suicidal ideation at the baseline and participants with suicidal ideation at the baseline

		**No suicidal ideation**				**Suicidal ideation**			
		
		**Escitalopram (n = 196)**		**Nortriptyline (n = 127)**		**Escitalopram (n = 254)**		**Nortriptyline (n = 219)**	
		**n**	**%**	**n**	**%**	**n**	**%**	**n**	**%**
Gender	Female	121	62	84	66	156	61	143	65
Marital status	Married/cohabiting	102	52	67	67	122	48	115	52
	Separated/divorced	43	22	24	19	55	22	45	21
	Single	48	25	28	22	67	26	50	23
	Widowed	3	2	8	6	10	4	9	4
Children	0	59	30	32	25	77	30	61	28
	1	63	32	46	36	83	33	58	26
	2	61	31	37	29	75	29	81	37
	3+	13	7	12	9	19	7	19	9
Occupation	Full-time work	72	37	38	30	99	39	75	34
	Part-time work	21	11	15	12	29	11	28	13
	Student	12	6	9	7	18	7	11	5
	Homemaker	7	4	7	5	7	3	13	6
	Retired	21	11	21	16	23	9	31	14
	Unemployed	63	32	37	29	78	31	61	28
Number of depressive episodes	1	77	39	29	23	82	32	70	32
	2	105	53	81	64	138	54	119	54
	3+	14	7	17	13	34	14	30	14
Number of suicide attempts		6	3	13	11	37	16	47	23
		Mean	SD	Mean	SD	Mean	SD	Mean	
Characteristics	Age	42.2	11.6	43.8	12.5	43.1	11.8	41.5	11.5
	Education (years)	12.1	2.9	12.1	3.2	12.1	3.3	12.1	3
	Age at onset	33.7	8.7	32	10.3	33	10.6	32.1	9.9
	Duration (weeks)	19.7	11	17.6	11.5	18.8	12.5	19.3	13.9
Baseline severity	Observed mood	0.8	0.6	0.7	0.5	1	0.6	1.1	0.6
	Suicidal score	0.05	0.4	0.05	0.4	1.3	0.5	1.3	0.5

SD = standard deviation.

### Suicidality-related adverse events

Among those reporting significant suicidal ideation at baseline, two women (44 and 34 years old) randomised to escitalopram were admitted to hospital owing to suicidal ideation during the course of the study. Among those with no significant baseline suicidal ideation, a 55-year-old woman randomised to nortriptyline committed suicide in the ninth week of treatment. In the following analyses, these subjects were considered as having an increase of more than 1 SD at the time of dropout. Based on a score of four on the third item of the HRSD-17, and as shown in Table 3, eight individuals reported a suicide attempt, all of them at baseline. Only one of them also reported a suicide attempt during the first week of treatment.

### Changes in the continuous suicidal score over the treatment period

**Figure 2:**
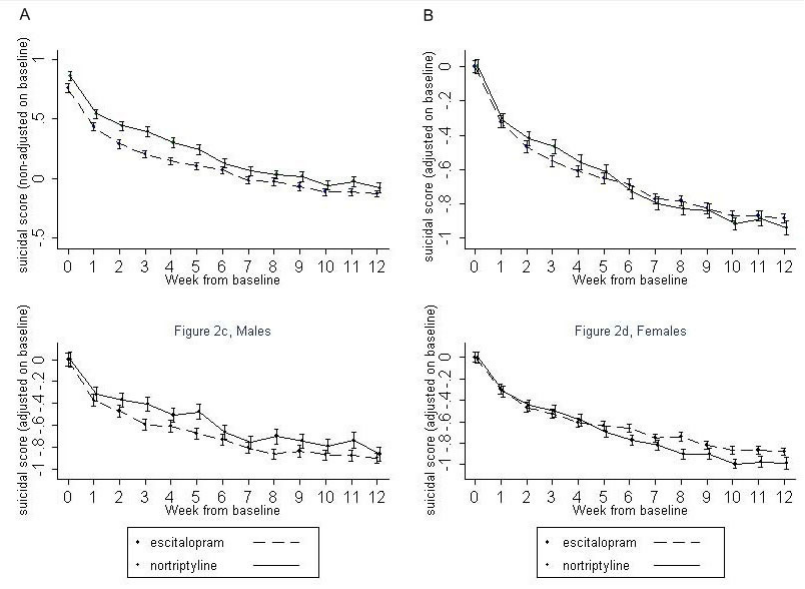
**Mean suicidal score unadjusted (a) and adjusted (b) from baseline score for escitalopram and nortriptyline for the 12 weeks of follow-up**. Panels **(c) **and **(d) **display the baseline-adjusted mean suicidal score for men and women, respectively, during the 12 weeks of follow-up.

Treatment with either drug was associated with a significant reduction in suicidal ideation over time (β = -0.48; z = -24.99; *P *< 0.0001) (Figure 2). Throughout the study, the mean suicidal score was higher in the nortriptyline-treated group (β = 0.14; z = 6.55; *P *< 0.0001). However, this difference was not significant after adjusting for the baseline suicidal score (*P *= 0.449). Age, gender or randomisation status had no effect on change in suicidal ideation over time (*P *= 0.659, *P *= 0.801 and *P *= 0.540, respectively). Married status was associated with lower suicidal scores (β = -0.15; z = -3.55; *P *< 0.0001).

### Increases in suicidal ideation score during treatment

**Figure 3:**
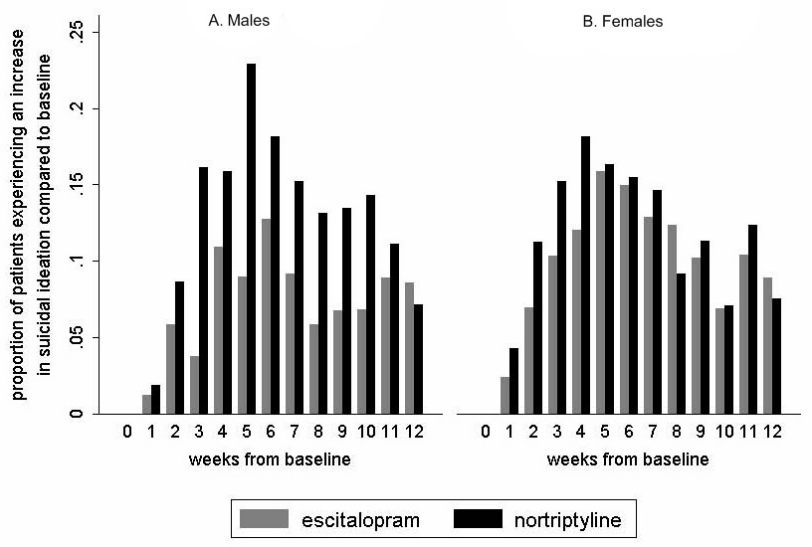
**Proportion of increases in suicidal ideation by study week among men (a) and women (b) for escitalopram and nortriptyline**.

In all, 254 subjects (31.9%) had an increase in suicidal ideation score of at least 0.5 SD at some point during the study. The highest rate was observed during the fifth week of treatment (15.5%). Age, gender, and interaction between these two variables did not predict an increase in suicidal ideation. In men and women combined, there was no association of either drug with increases in suicidal ideation and dose changes had no effect (Table 5). Random allocation was associated with increases in suicidal ideation (Table 5). Randomisation status was therefore used in all analyses as a covariate. Subjects who switched medication had a higher rate of increased suicidal ideation (Table 5). There was a gender by drug interaction (*P *< 0.0001) with a higher risk of increase in suicidal ideation among men taking nortriptyline compared to men taking escitalopram (OR = 3.51, 95% confidence interval (CI) 1.92 to 6.39, *P *< 0.0001) (Figure 3). Of men taking nortriptyline, 35.29% (n = 42/119) had an increase in suicidal ideation compared to only 23.70% (n = 41/173) of those taking escitalopram. This gender by drug interaction remained significant after adjustment for baseline severity of depression and severity of depression during the study. Severity of depression was strongly associated with a higher rate of increases in suicidal ideation (OR = 4.16, 95% CI 3.24 to 5.35 per standard deviation on observed mood, *P *< 0.0001). Younger age at onset and a higher number of depressive episodes also predicted increases in suicidal ideation (Table 5). In addition, increases in suicidal ideation were predicted by history of suicide attempts (OR = 3.59, 95% CI 2.31 to 5.58, *P *< 0.0001). Finally, retirement was found to be associated with increases in suicidal ideation (OR = 9.28, 95% CI 4.61 to 18.72, *P *< 0.0001).

**Table 5:** Results of the generalised linear latent and mixed model for TESI, TWOSI and 0.5 standard deviation (SD) increased

	**TESI**			**TWOSI**			**0.5 SD**		
	
	**OR**	***P *value**	**95% CI**	**OR**	***P*value**	**95% CI**	**OR**	***P*value**	**95% CI**
Age (continuous)	2.04	0.079	0.92 to 4.5	0.91	0.425	0.71 to 1.16	1.33	0.317	0.76 to 2.34
Age (categorical)	6.57	0.149	0.51 to 36.75	0.83	0.629	0.39 to 1.77	1.14	0.452	0.81 to 1.59
Gender	1.76	0.178	0.77 to 3.98	2.33	0.188	0.66 to 8.24	1.91	0.287	0.58 to 6.33
Randomisation status	1.16	0.667	0.58 to 2.32	4.39	0.022	1.23 to 15.64	6.05	0.003	1.82 to 20.07
Drug (nortriptyline vs escitalopram)	1.57	0.431	0.51 to 4.85	1.05	0.797	0.71 to 1.59	1.01	0.947	0.73 to 1.39
Observed mood dimension	2.92	<0.0001	1.92 to 4.46	3.28	<0.0001	2.49 to 4.32	4.16	<0.0001	3.24 to 5.35
Age at onset	0.16	0.01	0.04 to 0.64	0.66	0.296	0.30 to 1.44	0.42	0.037	0.18 to 0.95
Number of episodes (more than 3)	9.7	0.031	1.23 to 76.2	4.17	0.172	0.54 to 32.13	11.05	0.022	1.41 to 86.93
Duration	0.95	0.291	0.86 to 1.04	1	0.325	0.99 to 1.01	1.01	0.065	0.99 to 1.02
Switchers	2.2	0.022	1.11 to 4.35	5.94	<0.0001	4.19 to 8.44	9.47	<0.0001	6.57 to 13.6
Increasing dose	2.03	0.92	0.44 to 9.48	1.31	0.269	0.81 to 2.11	1.42	0.184	0.85 to 2.37
Decreasing dose	1.29	0.84	0.18 to 4.29	0.63	0.567	0.13 to 3	1.05	0.95	0.24 to 4.51
History of suicide attempts	0.85	0.898	0.07 to 9.48	2.02	0.015	1.15 to 3.45	3.59	<0.0001	2.31 to 5.58
Marital status									
Married/cohabiting	4.66	0.017	1.32 to 16.42	0.73	0.285	0.41 to 1.29	0.79	0.723	0.23 to 2.81
Separated/divorced	0.24	0.065	0.05 to 1.09	1.24	0.516	0.64 to 2.42	0.81	0.771	0.19 to 3.44
Single	0.38	0.162	0.11 to 1.47	2.02	0.094	0.88 to 4.58	1.85	0.507	0.31 to 11.28
Occupation									
Full-time work	0.27	0.073	0.04 to 1.16	0.79	0.402	0.45 to 1.38	0.31	0.062	0.11 to 1.09
Part-time work	1.71	0.725	0.09 to 18.03	1.14	0.754	0.51 to 2.52	1.51	0.631	0.28 to 8.17
Unemployed	6.84	0.002	2 to 23.3	0.75	0.323	0.41 to 1.33	1.82	0.337	0.53 to 6.29
Retired	0.93	0.96	0.08 to 9.58	3.25	0.009	1.33 to 7.92	9.28	<0.0001	4.61 to 18.72
Student*	-	-	-	1.06	0.929	0.32 to 3.46	0.33	0.398	0.02 to 4.34
Children (yes vs no)	1.04	0.978	0.05 to 21.43	0.57	0.124	0.29 to 1.16	0.49	0.378	0.11 to 2.33
Education	1.12	0.504	0.79 to 1.59	0.95	0.264	0.87 to 1.04	0.91	0.358	0.76 to 1.11
Gender by drug interaction		<0.0001			<0.0001			<0.0001	
Drug among men (nortriptyline vs escitalopram)	9.83	0.01	1.72 to 56.27	2.47	0.004	1.34 to 4.56	3.51	<0.0001	1.92 to 6.39

^a^No students with TESI were observed.

CI = confidence interval; OR = odds ratio; TESI = treatment emergent suicidal ideation; TWOSI = treatment worsening suicidal ideation.

The results from the two sensitivity analyses were very similar to the whole sample; all parameter estimates were in the same direction and well within the 95% confidence intervals of the parameters derived from the whole sample analysis.

### Emergence of new suicidal ideation during treatment (TESI)

Just over 17% (n = 57) of the participants with no baseline suicidal ideation qualified for the definition of TESI at some point during follow-up. The highest rate of TESI (6.6%) was observed during the fifth week of treatment.

TESI was predicted by depression severity during follow-up (OR = 2.92, 95% CI 1.92 to 4.46 for 1 SD on observed mood, *P *< 0.0001), younger age at onset of depression (OR = 0.16, 95% CI 0.04 to 0.64 for 1 SD increase in age at onset, *P *= 0.01), higher number of previous depressive episodes (more than two previous episodes: OR = 9.7, 95% CI 1.23 to 76.2, *P *= 0.031), unemployment (OR = 6.84, 95% CI 2 to 23.3, *P *= 0.002) and being married (OR = 4.66, 95% CI 1.32 to 16.42, *P *= 0.017). Drug, age, gender, randomisation status, history of suicide attempts, education or children did not significantly predict TESI. There was a significant gender by drug interaction (*P *< 0.0001), explained by a higher risk of TESI in men taking nortriptyline compared to men taking escitalopram (OR = 9.83, 95% CI 1.72 to 56.27, *P *= 0.01). In all, 20.93% (n = 9/43) of men taking nortriptyline had TESI compared to 10.67% (n = 8/75) of those taking escitalopram. This interaction just missed the threshold for statistical significance after adjustment for baseline severity of depression and severity of depression during the study (*P *= 0.063). TESI subjects more often crossed over to a second course of medication (*P *= 0.022).

### Worsening of pre-existing suicidal ideation during treatment (TWOSI)

Of the 473 subjects who reported significant suicidal ideation at baseline, 197 (42%) qualified for TWOSI at some time during treatment. The highest rate of TWOSI (22%) was in the sixth week of treatment.

TWOSI was predicted by depression severity during the follow-up (OR = 3.28, 95% CI 2.49 to 4.32 for 1 SD increase in observed mood, *P *< 0.0001), history of suicide attempts (OR = 2.02, 95% CI 1.15 to 3.45, *P *= 0.015) and retirement (OR = 3.25, 95% CI 1.33 to 7.92, *P *= 0.009). Drug, changes in dosage, age, gender, age at depression onset, marital status, education or children did not significantly predict TWOSI. Randomisation status was significantly associated with TWOSI (*P *= 0.022). There was a drug by gender interaction (*P *< 0.0001) due to a higher risk of TWOSI in men taking nortriptyline than in men taking escitalopram (OR = 2.47, 95% CI 1.34 to 4.56, *P *= 0.004). A total of 43.4% (n = 33/76) of men taking nortriptyline had TWOSI compared to only 33.7% (n = 33/98) of those taking escitalopram. This interaction remained significant after adjustment for baseline severity of depression and severity of depression during the study. Subjects with TWOSI moved significantly more often to the second course of medication than participants without TWOSI (*P *< 0.0001).

### Self-reported suicidal ideation

As the BDI scale correlated the least with our composite suicidal ideation score (0.77) and this was the only self-report questionnaire, we repeated all analyses with this scale alone. The results were very similar to the main findings and all coefficients were in the same direction.

### Clinician-rated suicidal ideation

In order to compare our results to an established way of defining suicidal ideation, we repeated the analyses using the more stringent traditional cut-off of 2 on the third item of the HRSD-17. Although this analysis was slightly less efficient, all results were in the same direction with estimates within the 95% CIs of our main results and the gender by drug interaction remained significant (*P *= 0.045).

## Discussion

We here report time course and predictors of suicidal ideation during treatment with a TCA or an SSRI, evaluating a comprehensive set of predictors for both treatment-emergent and treatment-worsening suicidal ideation. Depression severity during the follow-up was the strongest predictor of both TESI and TWOSI. A drug by gender interaction indicated a higher risk of suicidal ideation during treatment with nortriptyline among men irrespective of the presence of suicidal ideation at baseline. This latter result was confirmed in a sensitivity analysis restricted to randomly allocated subjects.

Although it is reasonable to assume that antidepressants reduce suicidal behaviours by reducing depressive symptoms, it has also been suggested that in some cases suicidal thoughts and behaviours might be induced by antidepressant treatment. Several studies and meta-analyses found an increased risk of suicidal behaviours in adults treated with SSRIs compared with placebo and/or TCAs [4,5,18,27-30], but several other reports found no differences in suicidal behaviours between TCAs and SSRIs in adults [4,13,14,17,31-33].

In the GENDEP trial, suicidal ideation markedly decreased during treatment with either nortriptyline or escitalopram, with no overall difference between the two drugs. However, nortriptyline was associated with a higher rate of both TESI and TWOSI in men. Few studies to date have compared the impact of an SSRI and a TCA on suicidal ideation and none has considered moderation by gender [32]. The IRT suicidal score provided a sensitive measure to investigate this issue and to confirm that antidepressants are not only effective in reducing depressive symptoms but also in reducing suicidal ideation. These findings are in agreement with most epidemiological studies showing that mortality by suicide declines when antidepressant use increases [34-36]. The GENDEP results support a beneficial effect of antidepressants on suicidal ideation in adults and should encourage clinicians to prescribe antidepressant medication in patients with suicidal ideation.

One reason why the GENDEP results may differ from some previous studies is the fact that nortriptyline has a predominantly noradrenergic action compared to other TCAs. As overactivity of the noradrenergic system is associated with anxiety and agitation, the increase in noradrenergic transmission induced by nortriptyline may drive the suicidal risk [37,38]. It has been suggested that suicidal ideation is more common in agitated and irritable types of depression [39]. Therefore, it is possible that nortriptyline may induce or worsen suicidal thoughts in some male subjects possibly through an induction of this more agitated type of depression. The drug effect in GENDEP was concentrated in depressed men who suffer from irritability, anger and aggressive behaviours more often than women and may therefore be vulnerable to the enhancement of noradrenergic transmission. Interestingly, the effect of a genetic polymorphism in CREB1 on anger-related symptoms and on treatment-related suicidal ideation is also specific to male gender [12].

Another possibility is that the increased risk of suicidal ideation simply reflects a lower efficacy of nortriptyline on mood symptoms [21]. We found that participants with TESI and TWOSI suffered from more severe depression, had a younger age of onset and a larger number of depressive episodes. This is consistent with several other studies [9,11,13,40]. Perlis *et al*. [11] showed that participants with TESI from the Sequenced Treatment Alternatives to Relieve Depression (STAR*D) study responded less to antidepressants and had a more severe form of depression, and concluded that emergence of suicidal ideation may be a surrogate marker for lack of improvement. This concordant finding in the two largest antidepressant-treated samples to date justifies the interim conclusion that TESI and TWOSI are associated with an increase, or lack of improvement, in depressive symptomatology. Our finding that TESI and TWOSI may also be associated with emergence of psychomotor activation triggered by noradrenergic antidepressants leading to suicidal ideation remains to be evaluated in future studies.

The rates of TESI and TWOSI in GENDEP were higher than those reported previously [9-13,41]. This could be due to several reasons. First, the use of three measures to calculate the suicidal score has increased the sensitivity to detect suicidal thoughts as compared to studies that only used one scale. It could be argued that our IRT-derived suicidal ideation estimate encompasses too broad a range of suicidal ideation. However, the rate of TESI was very similar when the traditional threshold of 1 on the BDI was used to define suicidal ideation [11-13]. Importantly, results regarding predictors of TESI and TWOSI proved robust to more stringent thresholds for suicidality in sensitivity analyses. Second, the high rate of suicidal ideation could reflect the higher severity of depression in the GENDEP sample: the average baseline HRSD-17 score was 21.7 in GENDEP compared to 19.2 in STAR*D. However, comparisons across studies should be interpreted with caution, as the methodology and analysis differed significantly.

It has been suggested that the highest risk of suicidal behaviour is in the initial few weeks or even days of treatment with antidepressants [11,13,14,42]. Our findings do not support this notion and demonstrate that TESI and TWOSI were relatively evenly distributed over the 12 weeks of follow-up. This is of particular concern, as it shows that there is little reason for either intensive monitoring over the first weeks, or for complacency later in the course of treatment, which could be an interpretation of current clinical guidelines [7]. This intriguing result could be explained by the fact that we did not restrict our analyses to the emergence of suicidal ideation in individuals who reported no suicidal ideation at baseline but extended them to the worsening of existing suicidal ideation during treatment with antidepressants. Moreover, in the GENDEP study each participant was evaluated each week for 12 weeks giving us the opportunity to examine the time course of changes in suicidal ideation and thus to highlight TESI and TWOSI even in the later weeks. In agreement with our results, Zisook *et al*. [15] in a recent analysis of the STAR*D data also found that increase in suicidal ideation were not confined to the first weeks but often occur later in the course of the treatment.

We found that prior suicide attempts, unemployment or retirement were associated with TWOSI and TESI. These are known predictors of suicidal behaviours [33,43,44]. Surprisingly, married status was associated with a higher risk of TESI. However, concordant with our findings, a recent study looking at risk factors for suicide among psychiatric patients found that marriage increased the suicide risk [44].

### Strengths and limitations

GENDEP is the largest clinical trial to date comparing a TCA with an SSRI and provides sufficient power to detect events such as emergence or worsening of suicidal ideation. Another advantage of the present study is that the evaluation of suicidal ideation did not rely on a single rating scale and was able to integrate self-report with clinician evaluation. This reduces false positives and false negatives.

As our study is not placebo controlled, we are not able to distinguish specific effects of SSRIs or TCAs from placebo effects or natural fluctuations in the course of depression. The improvement in suicidal ideation could, therefore, also reflect a natural course with a diminution of symptoms in participants with suicidal ideation rather than a specific effect of drugs. An untreated or placebo-treated control group would be required to separate specific and non-specific effects. However, the absence of placebo allowed inclusion of more severe patients and individuals who would not have agreed to participate in a placebo-controlled study, thus increasing the generalisation of our findings to treatment seeking depressed individuals. Furthermore, the random allocation of a large proportion of participants to escitalopram or nortriptyline allows an unbiased comparison of the two antidepressants.

The fact that GENDEP was an open-label study might have influenced the outcome by modulating patient expectations. Participants' knowledge of being allocated to nortriptyline, an 'old antidepressant', could have led to less positive outcome expectations compared to those allocated to escitalopram, 'a new antidepressant'. However, this is unlikely to explain a drug difference that was found specifically in men, with no effect across the whole sample. We therefore believe that a biological explanation of such differential effect is more plausible.

Only one individual reported a suicide attempt while undergoing treatment in the GENDEP study. This low rate is in agreement with STAR*D results [9] and precluded investigating predictors of suicide attempts during antidepressant treatment. A synthesis of multiple studies is needed to explore determinants of actual suicide attempts during antidepressant treatment.

Another limitation is that our results could not be generalised to other forms of suicidal behaviours such as suicide attempts and suicide completion as the relationship between these phenomena has been shown to be imperfect [45].

Finally, the current study did not take into account the influence of alcohol or substance use disorders as severe forms of these constituted exclusion criteria in GENDEP.

## Conclusion

Both SSRIs and TCAs were associated with a significant reduction in suicidal ideation. The present analyses clearly indicate that TESI and TWOSI are associated with severe forms of depressive disorder and poor treatment response. Clinicians should be aware that TCAs are associated with at least equal risk of treatment-related suicidal ideation as SSRIs. Retired or unemployed men and those with a history of past suicide attempts are at particular risk of worsening in suicidal ideation and should be carefully monitored. The monitoring of suicidal ideation should not be restricted to the early phases of treatment.

A classification of suicidal ideation during antidepressant treatment is proposed, taking into account all subjects in the analyses with either *de novo *emergence of, or worsening in, suicidal ideation. The clinical predictors of TWOSI and TESI were similar, indicating that increase in suicidal ideation, irrespective of its presence at baseline, is one phenomenon and should be investigated as such.

## Competing interests

NH has participated in clinical trials sponsored by pharmaceutical companies including GlaxoSmithKline and Lundbeck. WM, AZ, AEF, PMcG and KJA have received consultancy fees, and honoraria for presentations and participating in expert panels from pharmaceutical companies including Lundbeck and GlaxoSmithKline. The other authors declare no competing interests.

## Authors' contributions

NP and RU contributed equally to this paper. NP analysed and interpreted the data, drafted and revised the manuscript, and finalised it for publication. RU contributed to acquisition, analysis, and interpretation of data, assisted with drafting of the manuscript, manuscript revisions, and approved the final version. KA as joint principal investigator of GENDEP with PM conceived and designed the study, supervised the acquisition of data across all the centres, assisted with data analysis and interpretation, manuscript drafting, revision, and approved the final version of the manuscript for publication. AF contributed to the study conception and design, supervision and acquisition of data, and manuscript review and final approval. The following, Lead Investigators and researchers at their respective recruiting centre, contributed to the study design, acquisition of data, and manuscript review and final approval: AM, MR, JS, OM, LJ, NH, JH, AS, WM, AZ, DS, AP, CG, CG, JG, DC, BG and AE.

## Pre-publication history

The pre-publication history for this paper can be accessed here:


